# Incomplete Milking in Early Lactation Does Not Affect Dairy Cows Resting Behaviors: Results from a Randomized Controlled Trial

**DOI:** 10.3389/fvets.2017.00066

**Published:** 2017-05-08

**Authors:** Catarina Krug, Trevor J. DeVries, Jean-Philippe Roy, Jocelyn Dubuc, Simon Dufour

**Affiliations:** ^1^Département de pathologie et microbiologie, Faculté de médecine vétérinaire, Université de Montréal, Saint-Hyacinthe, QC, Canada; ^2^Department of Animal Biosciences, University of Guelph, Guelph, ON, Canada; ^3^Département de sciences cliniques, Faculté de médecine vétérinaire, Université de Montréal, Saint-Hyacinthe, QC, Canada

**Keywords:** dairy cattle, animal welfare, resting behavior, data logger, incomplete milking

## Abstract

The objective of this study was to investigate the effect of incomplete milking during the first 5 days of lactation on the resting behavior of commercial dairy cows. The hypothesis was that the elevated intramammary pressure resulting from milk retained in the udder in incompletely milked cows could lead to a change in lying behavior. This study was a randomized controlled trial in which cows from two farms were randomly allocated into a treatment (*n* = 18) or a control group (*n* = 14). Cows in the treatment group were milked incompletely (10–14 L/days) during the first 5 days of lactation, while cows in the control group were milked as usually done on farm. Resting behaviors were recorded with a data logger. Linear mixed models were used to quantify the effects of treatment group on three dependent variables measured between 2 and 14 days in milk: daily duration of lying time (h/d), lying bout frequency (bouts/day), and mean duration of lying bouts (min/bout). There was no significant effect of treatment on lying time. However, the effect of treatment on frequency of lying bouts and on mean lying bout duration varied by parity level. Incompletely milked cows in second parity had a higher number of lying bouts (11.9 vs. 9.2 bouts/day) and shorter mean lying bout duration (57.8 min/bout vs. 66.7 min) than control cows. In third parity or more, the opposite happened. Therefore, our results suggest that an incomplete milking may be slightly problematic for second parity cows and, possibly, slightly beneficial for older cows. Whether the differences observed resulted from a biologic process (discomfort due to the incomplete milking) or from random error will have to be determined by future research.

## Introduction

Milking cows incompletely in early lactation is a novel way to reduce the negative energy balance and its detrimental effects in dairy cows (1). However, reducing the volume of milk harvested might potentially be associated with a sustained udder distention, especially in high producing cows, which could lead to a modification of the cow’s lying behaviors. Unfortunately, there are currently no published studies on this topic.

Internal or external challenges that lead to poor animal welfare often produce differences in cows’ behavioral activities, including resting behavior (2). For instance, a study by Österman and Redbo (3) showed that cows milked twice a day vs. cows milked three times per day had higher number of lying bouts of shorter duration and fewer long lying bouts 4 h before milking. Such difference in behavior was hypothesized by these authors to be caused by pain due to udder distension. However, welfare was not impaired by a lower milking frequency in other studies. For example, two studies (4, 5) reported that cows milked once a day had similar lying times and improved hoof health and locomotion score compared to cows milked twice a day. However, in these studies, cows were not assessed in early lactation, when milk yield is increasing. In another study (6), cows milked once a day had higher udder firmness, but similar grazing activity and a tendency for longer lying times compared to cows milked twice a day.

Work conducted at dry off may be useful in determining the potential impacts of incompletely milking cows in early lactation. For example, Zobel et al. (7) presented a review on the effects of abrupt cessation of lactation on animal welfare. According to these authors, the elevated intramammary pressure resulting from milk retained in the udder after milking cessation could lead to tissue damage and pain. The sum of articles reviewed in that paper, however, did not lead to a conclusive answer regarding changes in lying behavior following abrupt cessation of milking at drying off, and the authors suggested that level of milk production at dry off should be considered when conducting such analysis.

For the current study, our hypothesis was that the elevated intramammary pressure resulting from milk retained in the udder in incompletely milked cows could lead to a measurable change in resting behavior compared to cows milked completely. Therefore, the objective of the current study was to investigate the effect of an incomplete milking during the first 5 days of lactation on daily duration of lying time, lying bout frequency, and mean duration of lying bouts of commercial dairy cows up to 14 days in milk (DIM).

## Materials and Methods

### Sample Size Estimation/Power

The current study was initiated following discussions with producers participating in a larger randomized controlled trial (RCT). Producers were concerned about the potential discomfort of the treatment procedure for their cows. With the larger RCT already ongoing, a limited number of cows were available for studying impact of treatment on resting behaviors. We expected to be able to recruit approximately 32 cows (16 in each group) before the end of the study, which would contribute to around 448 daily observations (32 cows multiplied by 14 days). Rather than a sample size estimation, we estimated the minimal difference that could be detected with the available sample size using SAS power procedure. A power of 90% and a level of confidence of 95% were used. For lying time, assuming a SD of 1.3 h/day in the control group, it was deemed possible to detect with a power of 90% a difference ≥0.4 h/day between treatment groups. For lying bouts frequency, with an expected SD of 3.8 bouts/day in the control group, it was deemed possible to detect a different of at least 1.2 bouts/day between treatment groups. For mean lying bout duration, with an expected SD of 12 min/bout, it was judged possible to detect a difference of at least 3.7 min/bout between treatment groups.

### Animals and Treatments

This study was part of a larger RCT that was conducted on multiparous cows from a convenient sample of 13 commercial dairy farms in the province of Quebec, Canada. An article describing this larger RCT is in preparation (Morin et al., personal communication). The eligibility criteria for these farms included: being enrolled in a Dairy Herd Improvement program, having a milking system that allows measurement in real time of the volume of milk harvested from the cow, having computerized records of disease, having at least around 70 multiparous cows calving per year, and being willing to apply the methodology necessary for the study and to share their herd records with the research group. The study protocol was accepted by the Animal Ethics Committee of the Université de Montréal (rech-1701). For this RCT, all multiparous cows, in the study herds, calving during the 14-month period comprised between January 2013 and March 2015 were recruited. For the current study, cows from two of the participating herds that calved in the last 5 months of the RCT (i.e., from October 2014 to February 2015) were recruited. In these two herds, cows were housed in free stall barns (mattress-based stalls covered with wood shavings as bedding). Herds were milked twice (04:00 and 16:00 h; herd A) and three (04:30, 12:30, and 20:30 h; herd B) times a day. Herd A had a mean number of 68 milking Holstein cows and a mean 305-day milk yield of 10,091 kg per cow whereas herd B had a mean number of 189 milking Holstein cows and a mean 305-day milk yield of 9,155 kg per cow. During the study, cows were randomly allocated to a treatment or a control group using a random number generator. Cows in the treatment group were milked incompletely during the first 5 DIM: 10 L on day 1, 10 L on day 2, 10 L on day 3, 12 L on day 4, and 14 L on day 5. The decision on the quantity of milk withdrawn per day was based on the study from Carbonneau et al. (1). Cows in the control group were milked completely, as usually done on these farms. Because treatment influenced how cows were milked, dairy producers could not be blinded to the group allocation (treatment or control).

### Animal-Based Measures

Parity and calving date were obtained through farm records. Resting behavior was recorded with Hobo Pendant Acceleration data loggers (Onset Computer Corporation, Bourne, MA, USA) validated by Ledgerwood et al. (8). The data logger was installed 1 week before expected calving and replaced every week until the end of the second week of lactation. The device was set to record g-force and slope of the *x, y*, and *z*-axes in a scale of −3.2–3.2 at intervals of 60 s (9). The data loggers were attached with bandage to the left hind leg above the metatarsophalangeal joint of cows for easy access in the milking parlor during the following weeks. The three axes were drawn on the exterior of the data logger and, when attached to the leg, the data logger was placed with the illustrated *x*-axis parallel to the ground pointing to the head of the animal, the *y*-axis perpendicular to the floor pointing to cow’s back, and the *z*-axis parallel to the floor pointing to the lateral of the cow (9).

### Data Management and Statistical Analyses

**Figure 1:**
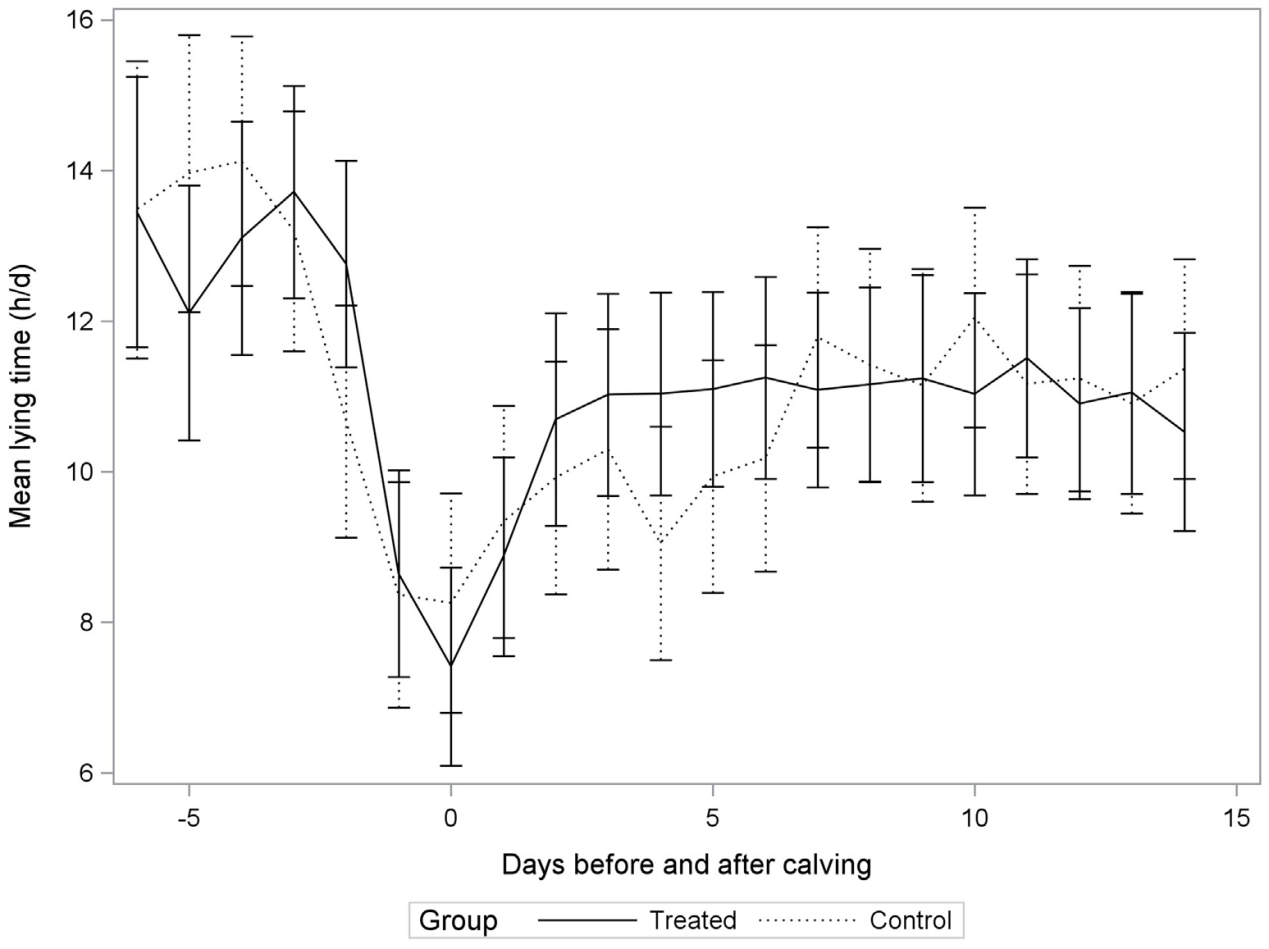
**Non-adjusted least square means for lying time (h/day) in incompletely milked (Treated) cows and control cows (Control) in a randomized controlled trial conducted on 32 dairy cows from two commercial herds**.

To extract the data from the data logger, the Onset Hoboware Pro Software (Onset Computer Corporation, Bourne, MA, USA) was used. Data were then imported as comma separated values files in SAS software (version 9.4, SAS Institute Inc., Cary, NC, USA) to be edited using the standard operating procedures described by the University of British Columbia (9). Three outcomes (daily duration of lying time, h/d; lying bout frequency, bouts/day; and mean duration of lying bouts, min/bout) were computed for each cow-day of observation. These outcomes were considered as the dependent variables in this study. Since there is usually a drop in lying time around calving (see Figure 1, for example), only observations from 2 to 14 DIM were used in the models. The predictor of interest in the current study was treatment group (i.e., incomplete vs. complete milking). Researchers assessing the outcome were not blinded to treatment allocation.

Prior to modeling, quantitative variables were tested for normality by visual inspection of histograms. Variables were then screened individually for their association with the three dependent variables using linear mixed regression models using the MIXED procedure of SAS 9.4. Models were then developed to investigate conditional associations. The 13 daily observations were clustered by cow; therefore, a cow random intercept was used in the models; herd was included in the models as a fixed effect to control for clustering of cows by herd. Furthermore, a treatment × DIM interaction term was forced into the models to capture the daily variance and to investigate the effect of the treatment for each day of observation. In these models, DIM was treated as categorical variable (i.e., day 2 to day 14). The Tukey adjustment was used to account for multiple comparisons.

There was an equal proportion of cows in parity 2 and parity ≥3 (16 cows in each category). The distribution of parity, however, was different between treatment groups (*P* = 0.03); with 12 second parity cows (67%) in the treated group and four second parity cows (29%) in the control group. Consequently, parity was kept as a fixed effect in all models to account for confounding by parity of the relationship between treatment and resting behavior. By keeping parity in each model, the reported effect of treatment on each outcome can then be interpreted as the effect of treatment on resting behavior if parity level had been held constant (i.e., if parity level was the same in treated and control cows). The linear mixed models were as follows:

(1) $$\begin{array}{c} {\text{RestBv}}_{ij}=\;\beta_{0}+\;\beta_{1}{\text{Tx}}_{j}+\;\beta_{2}{\text{Herd}}_{j}+\;\beta_{3}\text{Parit}y_{j}+\;\beta_{4}{\text{DIM}}_{ij}+\;\beta_{5}{\text{Tx}}_{j}*{\text{DIM}}_{ij}+\;v_{0j}+\;e_{0ij}, \end{array}$$

where RestBv*_ij_* is the predicted resting behavior (i.e., either daily duration of lying time, lying bout frequency, or mean duration of lying bouts), for the *i*th day from the *j*th cow; β_0_ is the intercept; β_1_ is the regression coefficient for treatment group; β_2_ is the herd fixed effect included to account for clustering of cows by herd; β_3_ is the effect of parity and is included strictly to account for confounding of the treatment effect by parity; β_4_ is the DIM effect; β_5_ is the treatment × DIM interaction; and *v*_0_*_j_* and *e*_0_*_ij_* are the cow random intercept and measurement error term, respectively (all assumed to follow an approximately normal distribution).

Parity (categorized as parity 2 and parity ≥3) was tested as a potential effect modifier by adding the main term and an interaction term with treatment group in the models. Parity was retained as an effect modifier if the interaction term yielded a *P*-value <0.20 on the *F* test. The interaction between parity and DIM was also tested and retained if the interaction term yielded a *P*-value <0.20 on the *F* test. Residuals were visually examined for each model to evaluate normality using quantile–quantile plot and histogram of residuals. Assumption of homoscedasticity was assessed visually using plot of the residuals against predicted values.

## Results

Data loggers were attached to a total of 38 cows, but 6 cows (4 from control group and 2 from treatment group) were excluded due to abnormal data records indicating misplacement of the logger (*n* = 3), or due to sickness/death (*n* = 3). In the end, 32 cows (22 from herd A and 10 from herd B) had usable resting behavior data: 14 were from control group and 18 from treatment group. Daily data were missing for some cows due to logger failure, therefore, out of a potential number of 448 cow-day observations, there were 331 usable cow-day observations and a mean number of 10.3 days of observation per cow.

**Figure 2:**
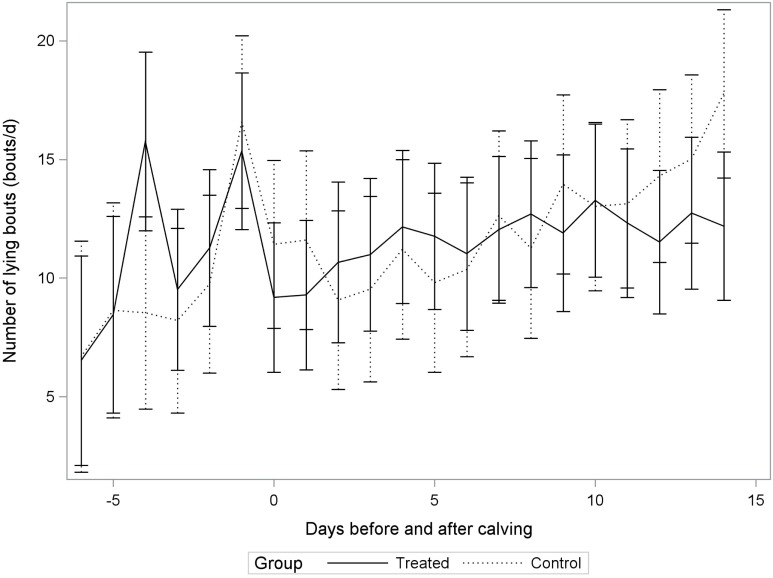
**Non-adjusted least square means for lying bout frequency (bouts/day) in incompletely milked (Treated) cows and control cows (Control) in a randomized controlled trial conducted on 32 dairy cows from two commercial herds**.

**Figure 3:**
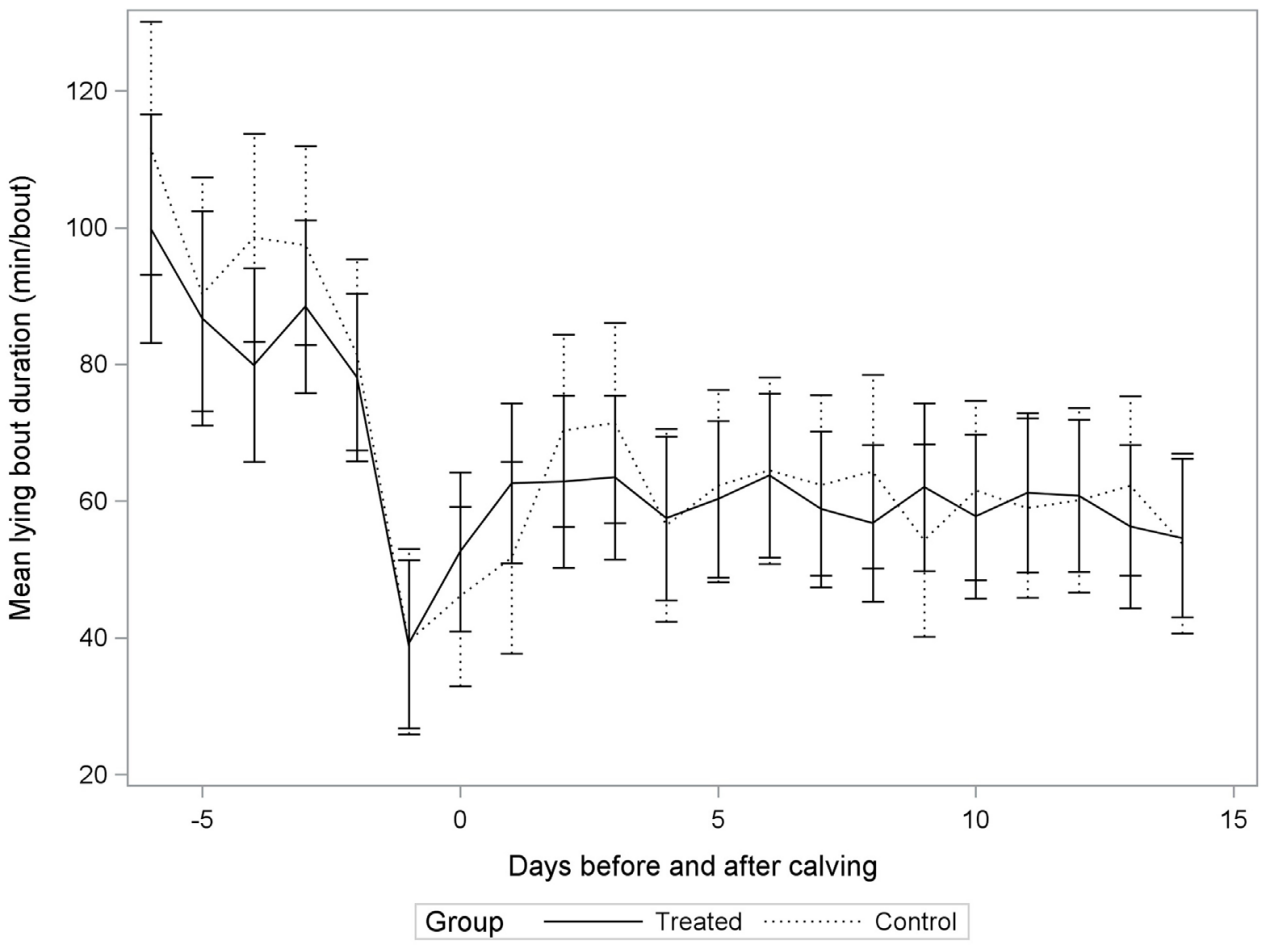
**Non-adjusted least square means for mean lying bout duration (min/bout) in incompletely milked (Treated) cows and control cows (Control) in a randomized controlled trial conducted on 32 dairy cows from two commercial herds**.

The average daily lying time was 11.0 ± 2.2 h/day, with an average frequency of 13.1 ± 6.4 bouts/day, and a mean lying bout duration of 56.9 ± 18.1 min/bout when considering only the 2–14 DIM period. Figures 1–3 illustrate distributions of non-adjusted least square means for lying time (h/day), frequency of lying bouts (bouts/day), and mean lying bout duration (min/bout), for control and treatment groups between 6 days before calving and up to 14 days after calving.

The treatment–lying time relationship varied as function of DIM (Table 1; Figure 1). However, after adjusting for multiple comparisons, there were no significant differences between treatment groups for none of the DIM. Lying times were, therefore, comparable between treatment groups throughout the 2–14 DIM period. The effect of treatment on lying time was not modified by parity level (*P*-value: 0.77).

**Table 1:** **Conditional association between predictors and daily duration of lying down (h/d) from 32 dairy cows (two commercial herds) enrolled in a randomized controlled trial; estimates were obtained using linear mixed regression models**.

Variable	Level	β	SE	95% CI	*P*-value^a^
Intercept		10.0	0.9	8.1, 11.9	
Treatment					0.75
	Control	Reference			
	Incomplete	0.6	0.9	−1.2, 2.4	
DIM					<0.01
	2	Reference			
	3	0.2	0.7	−1.1, 1.5	
	4	−1.0	0.6	−2.3, 0.2	
	5	−0.3	0.6	−1.5, 1.0	
	6	0.3	0.6	−1.0, 1.5	
	7	1.8	0.6	0.5, 3.0	
	8	1.4	0.6	0.1, 2.6	
	9	1.2	0.6	−0.0, 2.5	
	10	2.1	0.6	0.8, 3.3	
	11	1.1	0.6	−0.1, 2.4	
	12	1.1	0.6	−0.2, 2.3	
	13	1.0	0.6	−0.2, 2.2	
	14	1.4	0.6	0.2, 2.7	
Treatment × DIM					<0.01
	Incomplete × 2	Reference			
	Incomplete × 3	0.2	0.9	−1.5, 1.9	
	Incomplete × 4	1.4	0.8	−0.2, 3.1	
	Incomplete × 5	0.8	0.8	−0.9, 2.4	
	Incomplete × 6	0.4	0.8	−1.2, 2.1	
	Incomplete × 7	−1.3	0.8	−2.9, 0.3	
	Incomplete × 8	−0.8	0.8	−2.5, 0.8	
	Incomplete × 9	−0.6	0.9	−2.3, 1.1	
	Incomplete × 10	−1.7	0.8	−3.3, −0.0	
	Incomplete × 11	−0.2	0.8	−1.9, 1.4	
	Incomplete × 12	−0.7	0.8	−2.3, 0.9	
	Incomplete × 13	−0.5	0.8	−2.1, 1.2	
	Incomplete × 14	−1.5	0.8	−3.1, 0.1	
Parity					0.49
	2	Reference			
	3	0.5	0.7	−0.9, 1.9	
Farm					0.74
	2	Reference			
	1	−0.2	0.7	−1.6, 1.1	
Cow-level variance	–	3.0	–	–	–

*^a^Joint P-value obtained using an F test*.

*β, coefficient; CI, confidence interval; DIM, days in milk*.

When investigating lying bouts frequency, the effect of treatment did not vary as a function of DIM (Table 2; Figure 2), but it varied as a function of parity (*P*-value: 0.10; Table 2). For second parity cows, we observed, in incompletely milked cows, 11.9 bouts/day (95% CI: 9.3, 14.4) compared to 9.2 bouts/day (95% CI: 4.4, 13.9) for control cows. For ≥third parity cows, incompletely milked cows had 12.2 bouts/day (95% CI: 8.5, 15.9) compared to 15.4 bouts/day (95% CI: 12.7, 18.1) for cows in the control group. So treatment was associated with a higher number of lying bouts in second parity cows, while it was associated with a lower number of bouts in older cows.

**Table 2:** **Conditional association between predictors and lying bout frequency (bouts/day) from 32 dairy cows (two commercial herds) enrolled in a randomized controlled trial; estimates were obtained using linear mixed regression models**.

Variable	Level	β	SE	95% CI	*P*-value^a^
Intercept		5.0	3.0	−1.2, 11.2	
Treatment					0.89
	Control	Reference			
	Incomplete	4.6	3.2	−1.7, 11.0	
DIM					<0.01
	2	Reference			
	3	0.6	2.2	−3.7, 4.9	
	4	2.1	2.1	−2.1, 6.2	
	5	0.8	2.1	−3.4, 5.0	
	6	1.5	2.1	−2.6, 5.6	
	7	3.6	2.0	−0.4, 7.6	
	8	2.1	2.1	−2.0, 6.3	
	9	5.0	2.1	0.8, 9.1	
	10	4.0	2.0	−0.0, 8.0	
	11	4.2	2.0	0.2, 8.2	
	12	5.3	2.1	1.2, 9.4	
	13	6.2	2.0	2.2, 10.2	
	14	8.9	2.0	4.9, 12.9	
Treatment × DIM					0.13
	Incomplete × 2	Reference			
	Incomplete × 3	−0.1	2.8	−5.7, 5.5	
	Incomplete × 4	−0.4	2.8	−5.9, 5.1	
	Incomplete × 5	0.6	2.8	−4.9, 6.0	
	Incomplete × 6	−0.8	2.8	−6.2, 4.6	
	Incomplete × 7	−2.1	2.7	−7.3, 3.2	
	Incomplete × 8	0.2	2.8	−5.3, 5.6	
	Incomplete × 9	−3.7	2.8	−9.3, 1.8	
	Incomplete × 10	−1.2	2.7	−6.6, 4.2	
	Incomplete × 11	−2.3	2.7	−7.7, 3.0	
	Incomplete × 12	−4.2	2.7	−9.5, 1.2	
	Incomplete × 13	−3.9	2.7	−9.4, 1.5	
	Incomplete × 14	−7.2	2.7	−12.6, −1.8	
Parity					0.06
	2	Reference			
	≥3	6.3	2.8	0.9, 11.7	
Treatment × parity					0.10
	Incomplete × 2	Reference			
	Incomplete × ≥3	−5.9	3.6	−12.9, 1.1	
Farm					0.39
	2	Reference			
	1	1.5	1.7	−1.9, 4.9	
Cow-level variance	–	16.7	–	–	–

*^a^Joint P-value obtained using an F test*.

*β, coefficient; CI, confidence interval; DIM, days in milk*.

Similar results were obtained for mean lying bout duration; the relationship between treatment and lying bout duration did not vary as function of DIM (Table 3; Figure 3), but varied as a function of parity (*P*-value: 0.10; Table 3). For second parity cows, we observed 57.8 min/bout (95% CI: 49.9, 65.6) in incompletely milked cows, compared to 66.7 min/bout (95% CI: 52.1, 81.4) for control cows. For ≥third parity cows, incompletely milked cows had 60.9 min/bout (95% CI: 49.4, 72.4) compared to 51.8 min/bout (95% CI: 43.4, 60.2) for cows in the control group. So treatment was associated with shorter bouts in second parity cows, while it was associated with longer bouts in older cows.

**Table 3:** **Conditional association between predictors and mean lying bout duration (min/bout) from 32 dairy cows (two commercial herds) enrolled in a randomized controlled trial; estimates were obtained using linear mixed regression models**.

Variable	Level	β	SE	95% CI	*P*-value^a^
Intercept		79.3	9.1	60.7, 97.9	
Treatment					0.99
	Control	Reference			
	Incomplete	−16.8	9.6	−35.7, 2.1	
DIM					0.03
	2	Reference			
	3	−2.2	5.9	−13.8, 9.4	
	4	−15.7	5.7	−27.0, −4.4	
	5	−10.9	5.8	−22.2, 0.42	
	6	−8.0	5.6	−19.0, 3.1	
	7	−10.4	5.5	−21.2, 0.4	
	8	−8.4	5.8	−20.0, 3.0	
	9	−18.3	5.7	−29.6, −7.1	
	10	−10.6	5.5	−21.4, 0.2	
	11	−13.8	5.5	−24.6, −3.0	
	12	−13.0	5.6	−24.1, −1.9	
	13	−10.2	5.5	−21.1, 0.6	
	14	−18.6	5.5	−29.5, −7.8	
Treatment × DIM					0.55
	Incomplete × 3	2.9	7.7	−12.3, 18.1	
	Incomplete × 4	10.5	7.6	−4.4, 25.5	
	Incomplete × 5	8.5	7.5	−6.3, 23.3	
	Incomplete × 6	9.4	7.5	−5.4, 24.2	
	Incomplete × 7	6.1	7.3	−8.3, 20.5	
	Incomplete × 8	2.1	7.5	−12.7, 16.9	
	Incomplete × 9	18.3	7.6	3.4, 33.3	
	Incomplete × 10	5.6	7.4	−9.0, 20.2	
	Incomplete × 11	12.3	7.4	−2.2, 26.8	
	Incomplete × 12	11.0	7.4	−3.5, 25.6	
	Incomplete × 13	4.7	7.4	−9.9, 19.4	
	Incomplete × 14	10.5	7.4	−4.0, 25.0	
Parity					0.28
	2	Reference			
	≥3	−14.9	8.5	−31.6, 1.7	
Treatment × parity					0.10
	Incomplete × ≥3	18.1	11.0	−3.6, 39.7	
Farm					0.50
	2	Reference			
	1	−3.6	5.3	−14.1, 6.9	
Cow-level variance	–	166.55	–	–	–

*^a^Joint P-value obtained using an F test*.

*β, coefficient; CI, confidence interval; DIM, days in milk*.

## Discussion

To our knowledge, this is the first study investigating the impact of an incomplete milking during the first 5 days of lactation on resting behavior. The mean total lying time for cows was within the range of previously reported studies [9.3–13.9 h/day, DeVries et al. (10); 9.7–12.9 h/day, Westin et al. (11)]. Number of lying bouts and mean lying bout duration were also in agreement with other studies [29–115 min/bout, Tucker et al. (12); 6–20 bouts/day and 48–96 min/bout, Gomez and Cook (13)]. The population studied, therefore, appears to be comparable to that of other studies. In the current study, there were no differences in resting behaviors among groups for none of the DIM. So, in general, we could conclude that an incomplete milking during the early lactation does not lead to alteration of cows’ resting behaviors.

Similarly to what was observed by Calderon and Cook (14), lying time was decreased around calving, and then started increasing to reach a plateau around day 6. Lying time would usually be maintained for the remainder of the lactation after day 8 (14). In the current study, cows from the incomplete milking group seemed to reach this level of lying time earlier than conventionally milked cows, which could be interpreted as a positive effect of the incomplete milking. These differences, however, were not statistically significant and could, therefore, result simply from random error.

Although lying time was not altered by the milking protocol used, lying patterns differed by parity level. In second parity cows, we observed higher number of bouts and bouts of shorter duration in incompletely milked cows compared to control cows, while in third parity cows, incomplete milking resulted in a lower number of bouts and in longer mean lying bouts compared to the control cows. Whether these observed statistical interactions are truly the result of an existing biological interaction will have to be confirmed in future research using a larger sample size (and/or fewer degrees of freedom in the model). Nevertheless, we could hypothesize that a cow with a high number of bouts of short duration may be experiencing some level of discomfort. In fact, in a study from Siivonen et al. (15), cows with mastitis had lower lying times and a higher number of bouts of shorter duration per day. In that study, such a lying pattern was possibly caused by some level of discomfort due to inflammation of the udder. Therefore, our results suggest that an incomplete milking may not act in the same way for second compared to third parity cows and that it may be, somehow, slightly problematic for second parity cows and, possibly, slightly beneficial for third parity cows. These potential interpretations, however, must be considered cautiously.

The incomplete milking could also have altered cows’ metabolic status, which would, in turn, alter their feeding behaviors, and, consequently, their resting behaviors. The observed change in behaviors cannot, therefore, be directly interpreted as a sign of pain or discomfort. For instance, Carbonneau et al. (1) showed that the cow’s negative energy balance could be improved by reducing milk output during the first days of the lactation. We may hypothesize that an improved energy balance may have resulted in a reduced nutrient demands of the incompletely milked cows, and thus, in an alteration of their feeding behaviors. Thus, incompletely milked cows would have a greater amount of time that can be dedicated to activities other than feeding. Indeed, several researchers showed that cows with higher milk production have different resting patterns, mainly shorter lying times per day, than cows with lower milk production (16–18). This is probably a result of the higher energy requirements in cows that produce more milk, leading to an increased time standing while feeding at the feed bunk to meet those needs (10, 19). Tucker et al. (6) compared lying time from cows milked once (*n* = 20) and twice a day (*n* = 40) from 52 to 55 DIM and found that cows milked once daily had a tendency to spend more time lying down (9.8 h/day) than cows milked twice daily (8.3 h/day) in a 24 h basis. No differences in resting behavior were found in the 4 h before morning milking in that study.

In future research, recording resting and feeding behaviors altogether will possibly help understanding the effect of the milking protocol on the complete activity patterns of dairy cows. Furthermore, resting behaviors during the 4 h prior to milking could be specifically investigated, since milk accumulation is maximal during that period (20). In the current study, it could not be investigated because the exact time a cow was milked (or time at which she left the pen) was not recorded. Finally, it would also be valuable, in future research, to capture information regarding time spent standing in the stall and social behaviors (21).

There was no significant effect of treatment on lying time. However, the effect of treatment on frequency of lying bouts and on mean lying bout duration varied by parity level. Whether the differences observed resulted from a biologic process (pain or discomfort due to the incomplete milking) or from random error will have to be determined by future research.

## Ethics Statement

The study protocol was accepted by the Animal Ethics Committee of the Université de Montréal (rech-1701).

## Author Contributions

CK: conception or design of the work, data collection, data analysis and interpretation, drafting the article, final approval of the version to be published. TD: data analysis and interpretation, critical revision of the article, final approval of the version to be published. J-PR, JD, and SD: conception or design of the work, data analysis and interpretation, critical revision of the article, final approval of the version to be published.

## Conflict of Interest Statement

The authors declare that the research was conducted in the absence of any commercial or financial relationships that could be construed as a potential conflict of interest.
